# Loneliness and Absence in Psychopathology

**DOI:** 10.1007/s11245-023-09916-3

**Published:** 2023-04-25

**Authors:** Joel Krueger, Lucy Osler, Tom Roberts

**Affiliations:** 1 Department of Social and Political Sciences, Philosophy, and Anthropology, University of Exeter Amory, Rennes Drive, EX4 4RJ Exeter, UK; 2 School of English, Communication and Philosophy, Cardiff University, John Perceival Building, Cardiff, CF10 3EU Cardiff, UK

**Keywords:** Loneliness, Absence, Recognition, Psychopathology, Depression, Anorexia nervosa, Autism

## Abstract

Loneliness is a near-universal experience. It is particularly common for individuals with (so-called) psychopathological conditions or disorders. In this paper, we explore the experiential character of loneliness, with a specific emphasis on how social goods are experienced as absent in ways that involve a diminished sense of agency and recognition. We explore the role and experience of loneliness in three case studies: depression, anorexia nervosa, and autism. We demonstrate that even though experiences of loneliness might be common to many psychopathologies, these experiences nevertheless have distinctive profiles. Specifically, we suggest that: (i) loneliness is often a core characteristic of depressive experience; (ii) loneliness can drive, and even cement, disordered eating practices and anorectic identity in anorexia nervosa; iii) loneliness is neither a core characteristic of autism nor a driver but is rather commonly experienced as stemming from social worlds, environments, and norms that fail to accommodate autistic bodies and their distinctive forms of life. We aim to do justice to the pervasiveness of loneliness in many — if not all — psychopathologies, while also highlighting the need to attend to psychopathology-specific experiences of loneliness, agency, and (non-)recognition.

## Introduction

Like grief, love, happiness, and heartache, loneliness is a near-universal experience. Loneliness is widely discussed in literature, poetry, and religious texts, and is a focus of ongoing work in sociology, psychology, and neuroscience. In recent years, loneliness has also come to be regarded as an urgent public health concern. Given this wide interest in loneliness — and related themes like solitude, isolation, alienation, and togetherness — it is therefore surprising that philosophers have had little to say about it. When they do consider it, philosophers tend to frame loneliness in existential (e.g., Nietzsche, Heidegger, Sartre) or political (e.g., Arendt) terms (Seemann 2022). These approaches are valuable. But they lack a characterization of the phenomenological character of loneliness, what this condition *feels like* and how it shapes different ways of experiencing ourselves and our relationships — or lack thereof — with others.

Recent philosophical work (Roberts & Krueger 2021) develops an account of the phenomenological character of loneliness. Roberts and Krueger (2021) argue that loneliness is an “emotion of absence”: an affective state in which an agent becomes aware that something is missing and out of reach, either temporarily or permanently. What is absent, they argue further, are *social goods* — conversation, companionship, possibilities for self-development, evaluative and interpretive frameworks, and so forth — that accrue from close personal relationships. To be lonely is to experience an absence of the possibilities these goods afford and, accordingly, to feel one’s agency as a social being diminished.

In what follows, we build on this work. We turn specifically to the phenomenology of loneliness in psychopathology — experiential disturbances of how an individual relates to herself, others, and the wider world. Phenomenological psychopathology remains a lively and productive area of research. Within this work, much recent attention has been paid to aspects of social and emotional disturbances within different forms of psychopathology (e.g., Bortolan 2022; Fuchs 2013; Fuchs & Schlimme 2009; Maiese 2016; Ratcliffe & Stephan 2014; Sass & Pienkos 2013; Stanghellini et al. 2019; Trigg 2016). Nevertheless, we argue that the experience of loneliness within psychopathology remains undertheorized. This matters because understanding the character of social and emotional disturbances — features such as social withdrawal, alienation, affective flattening, feelings of disconnectedness, social anxiety, etc. — is not, in and of itself, sufficient to understand the structure and character of loneliness and how it shapes different self-world alterations. Attention to the distinctive place of loneliness within various psychopathologies can therefore provide a richer phenomenological map of the terrain of these disturbances. It also has practical import insofar as it impacts diagnostic and remedial strategies.

We first give a brief overview of the character and significance of loneliness before highlighting how loneliness relates to recognition and agency. Next, we consider loneliness in the context of psychopathology. By attending to experiences of loneliness in depression, anorexia nervosa, and autism, we illuminate experiential dimensions of these conditions that are often overlooked. Moreover, even though experiences of loneliness might be common to many psychopathologies, we show that these experiences nevertheless have distinctive profiles worthy of more focused attention.

Before we start, a note on the word “psychopathology”. This word traditionally refers to the study of mental disorders involving “unusual” or “abnormal” thought, experience, and behavior. The three cases we discuss in this paper — depression, anorexia nervosa, and autism — are usually included as examples of psychopathological disorders (they are all included in *The Diagnostic and Statistical Manual of Mental Disorders, Fifth Edition*). While we have followed tradition in using this term, we have some hesitations.

With the rise of the neurodiversity movement, there are increasing calls for autism to be viewed not as a medical condition but rather as a different form of life (e.g., Chapman 2019, Walker 2021). This movement is motivated, in part, by concerns about pathologizing autism. We will not enter this complex debate here. Nevertheless, in line with many contemporary autistic communities and advocates, we do not view autism as a psychiatric or medical “disorder” (for a fuller discussion, see Chapman & Bovell forthcoming). Our inclusion of autism here tracks historic categorizations and definitions; while problematic, they help us see how loneliness has been overlooked in relation to so-called “psychopathologies”, as well as the importance of turning philosophical attention to this issue. Relatedly, we will use words like “disturbance” and “anomalous” to be descriptively neutral characterizations of ways individuals become experientially unmoored from themselves and the world — and not, then, as evaluative judgements.

## The Character and Significance of Loneliness

Loneliness is linked with our basic human need for affiliation and mutual recognition (Cacioppo and Patrick 2008; Rochat 2009; Svendsen 2017). It arises when these needs are not met. Loneliness can have a particularly distressing and unpleasant character, as well as a significant impact on our health and wellbeing. Loneliness is now widely seen as a pervasive public health concern. It is linked, for instance, to early mortality, elevated blood pressure and risk of stroke, higher susceptibility to dementia and cognitive decline, and increased rates of depression and suicide (McHugh Power et al. 2018, pp. 219–220). The rise of loneliness and its connection with public health concerns has been a topic of much discussion within the past decade, particularly following extended periods of lockdown and social isolation during COVID-19 (O’Sullivan et al. 2021). However, despite this intense recent focus, there is no consensus about what loneliness consists in, exactly. It is defined differently across the humanities, social sciences, and medical literatures (McHugh Power et al. 2018, p. 220). And while philosophers have not (until recently) said much about loneliness, discussions abound in other disciplines — often with distinct characterizations of loneliness. This diversity is not just a conceptual matter; it potentially shapes diagnostic and remedial measures, too.

This is not the place for a comprehensive overview of different characterizations of loneliness.^1^ Instead, we highlight some core phenomenological features of loneliness that help clarify both its felt character and its impact on how individuals navigate the social world and connect (or fail to connect) with others. These features will, in turn, help further clarify experiences of loneliness in the context of certain psychopathologies.

### Loneliness, Affect, and Agency

It feels like something to be lonely. For many, loneliness has a particularly awful and unsettling character, which is why it can potentially be so devastating for those who experience it — particularly over long stretches of time. But loneliness is not just a subjective condition. As psychologists note, the term “loneliness” can pick out an objective condition, too (Ma et al. 2020; Tietjen and Furtak 2021). Someone can live an unusually solitary life largely devoid of social contact without missing the interpersonal connections and relationships many crave. This person may *be* lonely, objectively speaking, insofar as they live most of their lives apart from others. But they may not *feel* lonely. They may prefer solitude and silence and therefore lack the negative feelings that often attach to the objective condition of loneliness.

Distinguishing between loneliness as an objective and a subjective condition is important because the two kinds of loneliness are not reliably correlated (Vincent 2020). Solitary people don’t always feel lonely, whereas some hyper-social people do. These observations have led loneliness researchers to argue that loneliness is a state of *perceived* social isolation, and not simply *physical* isolation (Ozawa-de Silva and Parsons 2020).

Getting clear about the complex experience of loneliness, its first-personal or subjective character, is important. One reason is that diagnostic and remedial measures may vary considerably depending upon what we think loneliness consists in, exactly, and how we think about its felt character (Seemann 2022, p. 2). For example, if (subjectively) *feeling* lonely is sufficient for loneliness, simply highlighting all the reasons that one is, in fact, not (objectively) lonely — and thus denying the validity of an individual’s experience — will not be an effective remedial strategy. Moreover, reminding lonely people that “it’s only temporary” — as many well-intentioned intervention strategies do — brings little comfort or validation to those who are chronically lonely. The experience of loneliness is phenomenologically complex and diverse; loneliness comes in different forms and degrees of intensity and duration (Candiotto 2022; Kidd 2022). To better address loneliness, therefore, it is important to clarify its phenomenological character and structure.

We draw on a recent philosophical analysis of loneliness (Roberts and Krueger 2021) that regards loneliness as an emotional condition that has both world- and self-directed elements. Many emotions involve *objects* that are the target of that emotion: I am angry *because* a colleague uttered an unkind remark, happy *because* my child has taken her first steps, etc. However, loneliness is somewhat different. This is because it essentially involves an experience of *absence*. To be clear, other emotions such as grief or homesickness involve an experience of absence, too. But the phenomenological core of loneliness is this felt encounter with absence. To be lonely is to experience various social goods — companionship, moral support, physical contact, affection, sympathy, trust, romance, friendship, etc. — as both *absent* and as somehow *out of reach* or *unattainable*.^2^ In this way, loneliness has a two-part psychological structure. It consists of a directedness toward some desired social good(s), along with the recognition that the good(s) are out of reach, which gives rise to its distressing character.

One way to support this view is to note that emotional experiences of absence are not exclusive to loneliness (Roberts 2019). There are many instances where we have a pro-attitude toward some absent quality or thing while, at the same time, recognizing that it cannot be easily achieved, generated, or brought about. Consider homesickness. The experience of homesickness involves an acute longing for home — and its attendant comforts, familiarity, resources, and relationships — along with the awareness that, for whatever reason, home is out of reach. What is experientially salient in homesickness is the felt distance (geographical, physical, or psychological) that lies between the individual and the goods of home. Home therefore becomes experientially present via its absence. Other examples abound: unrequited love, say, or the experience of envy, nostalgia, or grief.

Again, in the case of loneliness, what is experienced as absent are a range of social goods we feel are needed to establish and maintain our most valued human relationships, develop our identity, and give meaning to the projects and values that organize our lives. The scope and scale of these felt absences are what make loneliness so painful. Thinking about loneliness this way shows that experiences of absence are closely tied to experiences of *access*. It’s not just that these goods are missing from an individual’s life the way, say, one might realize that a slice of cake is now absent because it was previously consumed and then forgotten. It’s that these goods are, for whatever reason, qualities or things that cannot be readily achieved or brought about. They are absent and, adding to our distress, *inaccessible*. In this way, the emotional experience of absence within loneliness — and its connection with experiences of access, or lack thereof — impacts our sense of *agency*. We can probably replace the slice of cake, or at least get something close enough that it satisfies our desire for something sweet. But it may be the case that — or at least *feel* like — we are powerless to alleviate our loneliness. We might know what would help: a romantic partner, having a deceased parent back in our lives, or developing richer and more authentic friendships. Nevertheless, when we are lonely, we feel unable to realize conditions that would help us satisfy these social goods.

These observations highlight another important point: the emotional experience of loneliness isn’t just about the world and others. It has a self-directed element, too (Roberts and Krueger 2021, p. 196). It is fundamentally tied to our sense of agency — our sense of what we can or cannot do — as well as our self-conception and self-regard. With respect to the latter, loneliness might lead to increased self-doubt and loss of confidence — for instance, if we feel increasingly unable to connect with others and negotiate the social world, that we have no valuable role to play in others’ lives or lack access to those with whom we might develop and maintain our identity as, say, a sports fan or as someone with particular religious or political beliefs. This diminishment of our self-regard is an important part of the character of loneliness and worthy of more consideration (see Bortolan 2022). However, we are here more interested in the former; that is, how the feeling of inaccessibility that is part of the experiential structure of loneliness shapes our sense of agency.

By “agency”, we simply mean the feeling of being able to do things, as well as the sense that certain possibilities are available to us. Like “loneliness”, the term “agency” has both an objective and a subjective side. It can objectively be true that I can do things like speak to strangers at a party, raise my hand to ask a question during class, walk down an unlit street at night, challenge the terse border control officer at the airport, dine in a fancy restaurant downtown, or refuse to answer a police officer’s questions. I may have the necessary bodily and psychological capacities. Nevertheless, I may *feel* that I lack agency in these contexts, which means that they are no longer experienced as salient possibilities. This diminished subjective sense of agency can be due to many things: e.g., shyness, social anxiety, illness, a lack of self-confidence, culture shock, disability, or the pressure of power structures or normative expectations governing that context (Carel 2013; Roberts and Osler 2023).

Crucially, this attenuated sense of agency and possibilities can also stem from a felt *lack of recognition*. By “recognition”, we mean the feeling of appearing in the world as a unique self able to connect with and be seen by others, and to participate with them in the construction of shared projects and values. As we’ll see, the feeling of recognition is important for understanding how we come to feel at home in the world, as well as for understanding some ways loneliness renders parts of the world inaccessible, including in cases of psychopathology.

## Loneliness, Recognition, and (Diminished) Agency

In order to develop these themes further, we now draw on Sarah Drews Lucas’ (2019) recent work on loneliness, appearance, and what she terms “ontological agency”. For Lucas, agency is tied to loneliness. More precisely, *absence* of agency, she argues, is the condition of loneliness. Lucas defines loneliness as non-appearance before others (ibid. p. 710). We experience loneliness because we feel that we are somehow not recognized by others, somehow not *seen*. This failure to be seen, in turn, diminishes our sense of agency and felt possibilities insofar as we feel unable to appear in the world before others as a unique self. An agent, Lucas tells us, “exercises her agency when she gives an account of her discrete, particular judgment as part of a worldly conversation” (Lucas 2019, p. 710). However, when, for whatever reason, she is unable to do this — e.g., she is subject to socioeconomic factors or political regimes that systematically exclude voices and selves like hers — she experiences this failure to sustain herself in the world and appear before others as a kind of loneliness. In this way, Lucas fruitfully highlights some ways that ontological and political registers of agency and (absent) recognition can shape experiences of loneliness.

While we will touch on some political aspects of loneliness and recognition later, these will not be our focus in what follows.^3^ Instead, we are concerned with the feeling that one is, or at least potentially *can be*, both present to and recognized by the world. Again, this is the feeling of how it is to encounter the world and others, the feeling of being a participatory member of the spaces and projects others occupy and create. It rests on the sense of *presence* — presence to the world, others, and the things and spaces that organize our shared lives. In the experience of loneliness, this sense of presence is diminished and one’s agency is felt to be attenuated. Parts of the world feel inaccessible because, for whatever reason, one does not feel seen or recognized.

To get an initial sense of how these different ideas are phenomenologically interrelated — before then turning to psychopathology — consider a simple example: the experience of being lonely at a party. Imagine starting a job in a new city, and a coworker invites you to her place for a social gathering. You arrive eager to get to know her better and make friends. But it soon becomes clear that you aren’t particularly welcome. Your new colleague gives you a cursory greeting before moving on, and the guests seem uninterested in talking to you. No one appears to acknowledge your presence. After some unsuccessful attempts to engage, you begin to wander awkwardly through that space, self-consciously sipping a drink while staring at a bookshelf and plotting a way to leave without drawing attention to yourself.

In this situation, you may begin to feel lonely. The conversation swirling around you may be lively, the music to your liking, and the overall atmosphere festive, buoyant, and expectant. But you aren’t part of it. You don’t feel seen, *recognized*. And this absence of recognition leads, in turn, to a diminished sense of agency. In a deep bodily way, you don’t know what to do with yourself. It’s not just that others aren’t talking to you or are expressing only a superficial interest in you. Their nonrecognition is expressed in a more subtle *nonverbal* manner. As James Jardine (2020) reminds us, recognition is not confined to speech acts: “it is often though non-linguistic forms of bodily expressivity — as enhancing or entirely replacing speech actions — that others impress upon us our visibility or invisibility in a social sense, since social statuses of this kind can be conveyed without any linguistic communication being necessary” (Jardine 2020, p. 311). Accordingly, in this scenario, the inter-bodily rhythms and dynamics that normally carry us through different situations (e.g., shared glances, looks, inviting facial expressions, friendly gestures, touches, conversation, laughter, etc.) by indicating what to do and feel and say and when to do it are missing. This absence of recognition makes it clear that we are not invited to inhabit and co-construct shared “we-spaces” that make us feel connected with one another (Krueger 2011). And without this social scaffolding, you may become suddenly aware of your gestures, posture, where you’re standing or how you move; you feel uncharacteristically stiff and self-conscious as you move through that uninviting space alone and unrecognized.

This example highlights the connection between loneliness and “social doubt” (Roberts and Osler 2023): a disruption of the smooth, flexible, and transparent (i.e., largely unthinking) way we normally relate to the social world and opportunities it presents in everyday life. In social doubt, our general attunement to the social world — an implicit sense of what to do, when and how to do it, etc. — is disturbed, and we feel this disturbance at the level of *bodily possibilities.* We feel a sense of disorientation, a sense of being bodily out-of-sync with that space and “stopped” by the indifference, inhospitality, and lack of recognition from others (Ahmed 2007). In other words, we experience something like what Carel (2013) characterizes as “bodily doubt”: an experience of “unreality, detachment, and estrangement” in which we feel alienated from our physical and social environments, and our natural trust in our body and its abilities “is displaced by feeling of helplessness, alarm, and distrust” (ibid. p. 184).

As we explore below, loneliness — arising from lack of recognition — can give rise to forms of bodily and social doubt, which are experiences of diminished agency. Again, within loneliness, these dimensions are phenomenologically interrelated, hence its complex structure. As Lucas helpfully reminds us, “[a]gency, then, is not the capacity to align action and intention; it is, rather, the constant capacity to appear as a unique self in the world” (Lucas 2019, p. 715). And when this capacity is withheld or impeded via a lack of recognition, we feel a sense of powerlessness and loneliness.

We have now introduced several different ideas. The key takeaway points are these: as an emotional experience of absence, loneliness isn’t just about the world and what’s missing from our relation to it (i.e., access to social goods and relationships). It has a self-directed aspect, too. Loneliness can disrupt our fundamental sense of being in the world in a deep and pervasive way, including our sense of spatial orientation, bodily movement, and possibilities for action, expression, and connection. In short, it can lead to a *diminished sense of agency*. And the concept of “recognition” is important here because it bridges these world- and self-directed aspects of loneliness while bringing additional phenomenological texture to this account. This is because loneliness involves a felt absence not just of social goods in some abstract sense but also concrete *reciprocity* — specifically, the interactive possibilities and collaborative forms of self-understanding and shared emotions that social goods afford. Reciprocity signals recognition.^4^ It’s how we develop and explore our agency and sense of self in collaboration with others. But in loneliness, possibilities for reciprocity are experientially present precisely *via their absence*. Additionally, highlighting the connection between recognition and loneliness illustrates how the world can be set up in ways that create *spaces of loneliness*, spaces that limit or withhold possibilities for recognition and thus empty of connection and reciprocity. This perspective highlights our *vulnerability* to the people, things, and places around us. With this background in place, we now turn to a more focused consideration of loneliness in psychopathology.

## Loneliness in Psychopathology

Psychopathology is traditionally described as the study of mental disorders. This characterization has generated descriptive and diagnostic approaches that focus on individuals and their psychology and brain function. However, within phenomenological psychopathology, increased attention has been paid to *social*, *emotional*, and *relational* aspects of mental disorders. These approaches see psychiatric disorders as involving not just symptoms or factors inside an individual’s brain or body, but also breakdowns of an individual’s wider relation to the people, things, and spaces around them (e.g., Aho 2019; Brenico and Bizzari 2022; de Haan 2020; Fuchs 2018; Kirmayer 2019; Krueger and Colombetti 2018; Maiese 2016; Roberts et al. 2019). For example, current psychiatric opinion views core disturbances of mood and affect in depression as arising from negative cognitions, self-evaluations, and emotions like anxiety, shame, and guilt. Accordingly, depression is something inside an individual’s head. However, a relational approach (e.g., Fernandez 2014; Fuchs 2013; Lenzo & Gallagher 2020; Osler 2021a, 2022; Ratcliffe 2015; Slaby 2013; Whiteley forthcoming) argues that depression arises not just from neurobiological or psychological factors, but also significant alterations of how an individual experiences their body, inhabits space, and shares emotions with others.

Despite this focus on social and emotional aspects of psychiatric disorders, experiences of loneliness have not widely been theorized as a discrete category of experience. For example, loneliness is given almost no attention in two recent major publications providing an overview of current research in philosophy of psychiatry and phenomenological psychopathology: the *Oxford Handbook of Philosophy and Psychiatry* (2013), and the *Oxford Handbook of Phenomenology and Psychopathology* (2019). Within the latter, loneliness does come up once, in a quote from the Dutch psychiatrist J. H. van den Berg who says that “loneliness is the nucleus of psychiatry” (van den Berg 1972, p. 105). But within these two major works, no attempt is made to think about loneliness on its own terms.

One reason for this neglect, we suspect, is that loneliness within psychopathology is subsumed by more general discussions of things like social withdrawal, social impairments, experiences of alienation, and other disruptions of an individual’s ability to connect with others. Loneliness, it may be assumed, therefore does not require its own conceptual or phenomenological analysis.

We think this is mistaken. In what follows, we defend this claim by considering loneliness in *depression*, *anorexia nervosa*, and *autism*. We explore how loneliness develops and is articulated in different ways within these three cases. These examples are illustrative because they involve different *forms* and *origins* of nonrecognition and diminished agency, which lead to distinctive experiential profiles of loneliness. While we will not go so far as to defend van den Berg’s claim that loneliness is the core or “nucleus” of psychiatry, we nevertheless argue that loneliness within psychopathology deserves more attention than it’s so far received. Particular attention to the place of loneliness within psychopathology is important for several reasons: it provides a richer phenomenological map of the terrain of self-world disturbances distinctive of these conditions; it highlights underexplored factors that shape their development and character (e.g., nonrecognition and stigmatization, the role of the built environment); it foregrounds both the pervasiveness of experiences of loneliness across various psychopathological conditions, while also highlighting differences in how and why loneliness is experienced in different disorders; and it has practical import insofar as it may impact diagnostic and remedial strategies.

### Loneliness and Depression

Depression is an affective disorder characterized by “depressive mood”. While the DSM notes that depression often can “cause significant distress or impairment in social, occupational, or other important areas of functioning” (American Psychiatric Association 2013, p. 163), some working in phenomenological psychopathology have recently argued that social disconnection is not simply a common side effect of depression but is rather a *core feature* of the disorder. Thomas Fuchs (2013), for instance, describes depression *as* a disturbance of smooth bodily interaction with others. He highlights how in depression one no longer experiences oneself as bodily attuned to or resonating with other people and, as such, this leaves the depressed individual feeling socially cut off and isolated. In a similar vein, Matthew Ratcliffe (2015) calls attention to the pervasive experience of being disconnected from others that characterizes much depressive experience. Ratcliffe emphasizes how the very possibility of entering into various interpersonal relations with other people is experienced as peculiarly “out of reach”.

David Karp’s description of the feeling of isolation in depression is notably reminiscent of our above characterization of loneliness as a feeling of certain social goods being painfully inaccessible: “Much of depression’s pain arises out of the recognition that what might make one feel better – human connection – seems impossible in the midst of a paralyzing episode of depression. It is rather like dying from thirst while looking at a glass of water just beyond one’s reach” (Karp 2016, p. 73). In depression, individuals often report feeling a diminished ability to enter into certain interpersonal relations in ways that are fulfilling and satisfying. To again quote Karp (2016, p. 59), this often results in a persistent feeling that other people are having “an easy time of it” socially, and a comparative sense of failure in one’s ability to smoothly engage in social interaction. Further compounding the experience of loneliness in depression is a commonly articulated feeling that other people simply cannot understand what one is going through. This feeling of being misunderstood does not simply refer to instances where someone mistakes or misinterprets what a depressed individual is doing or experiencing. Rather, it is a profound sense that *no-one is able* to understand their depressed experience, that others *cannot* understand their experience of the world as drained of connection, significance, or energy. In short, depressed individuals often report feeling something like a *lack of recognition*.

As such, we might think feelings of loneliness, and not just social disruption or impairment, are often a core feature of depressive experience — where social goods are experienced as absent and inaccessible, one’s own social agency is experienced as diminished, and a despair that other people are incapable of understanding what one is going through and, thus, precluding the very possibility of being recognized by others. This absence can be experienced as a kind of hollowness or emptiness, a feeling that one no longer can partake in the same social world that everyone else inhabits. When it comes to depression, then, loneliness does not seem to be an incidental feature but a core or even constitutive element of the experiential texture of what it is to be depressed.

Other forms of psychopathology also involve experiences of loneliness. Perhaps most obviously we might list psychopathologies that also involve various forms of social disruption at their core. Social anxiety, like depression, appears to have a close relationship with loneliness – where one’s desire to obtain certain social goods is thrown into uncertainty due to a perceived doubt in one’s own social competence and reception. In the next two case studies, though, we want to draw attention to the way in which loneliness features in both anorexia nervosa and autism but is not a core or constituent feature. This focus, we think, captures the prevalence of loneliness in many different psychopathologies while accounting for nuanced differences in how, why, and when loneliness is experienced.

### Loneliness and Anorexia Nervosa

Anorexia nervosa (AN) is an eating disorder characterized by self-starvation. Loneliness is not generally considered a core diagnostic feature of AN. However, many anorectics report being lonely. They feel stigmatized, stereotyped, and cut off from communities who can understand and support them — a lack of access, in other words, to the kinds of social resources, relationships, and recognition that others take for granted. Accordingly, they often develop various ways to address this loneliness, where these strategies feed into and shape their disordered eating practices and anorexic identity. Examining how loneliness develops and is dealt with in these contexts can therefore be illustrative in that it helps provide a richer phenomenological picture of the drive for recognition and belonging that motivates many of these practices — beyond simplistic caricatures of AN as a “desire for thinness” — and the ways that anorectics negotiate and sustain them.

The American Psychiatric Association classifies AN as involving self-starvation that leads to dramatic weight loss. According to current diagnostic criteria, someone can only be diagnosed with AN when their self-starvation leads to “significantly low body weight”. However, critics note that this approach is problematic for several reasons. One worry is that someone may already be a practicing anorectic before they meet a certain weight-loss threshold and therefore not satisfy current diagnostic criteria. This is particularly problematic since AN is typically characterized in relation to white aesthetics regarding body weight, shape, and size. These criteria may therefore exclude a significant portion of the population (Kendall 2020). Others have also criticized characterizations of AN that place a “desire for thinness” at their core for oversimplifying or overlooking the embodied, affective, and interpersonal dimensions of AN (e.g., Bowden 2012; Evans 2022; Legrand and Briend 2015; Osler 2021b).

Highlighting the centrality of loneliness within AN doesn’t directly address these concerns. But as we’ll see, it does reinforce the need to adopt phenomenologically richer *relational* and *multidimensional* approaches to AN and eating disorders more generally. These approaches move beyond individualistic perspectives — which focus on things like genetic or psychological predispositions, or disturbances of body-related perceptions and experiences — and instead adopt a more holistic view of how contextual and relational factors give rise to and support disordered eating (Osler 2021b; Osler and Krueger 2021).

AN can play different roles in people’s lives. For some, it is a way to achieve control and self-reliance or a means to find a sense of achievement and purpose. Others state explicitly that AN helps them cope with periods of loneliness, such as at the end of a relationship: “It is always there for me stopping me feeling alone” (Arkell and Robinson 2008, p. 653). Individuals might “retreat” into AN as a response to loneliness. AN can provide a sense of purpose or an effective way of distracting from other kinds of emotional or bodily pain (Osler 2021b).

Developing or slipping back into patterns of disordered eating can themselves provide comfort and familiarity during times of upheaval. This familiarity is sometimes expressed in terms of a personification of AN itself, with anorectic individuals referring to their disorder as “Ana”: “Unlike other illness categories, anorexia nervosa was transformed from a clinical entity into a friend: it became Ana, a comforter – especially during the early “honeymoon phase” of the disorder (Allison et al. 2012, p. 119). Loneliness, then, can be a trigger for AN.

At the same time, however, disordered eating practices can also feed into and intensify feelings of social isolation and loneliness. Practically speaking, it can make it difficult for anorectic individuals to participate in events that are organized around food, leading to the avoidance of certain kinds of social activity and spaces (Levine 2012, p. 244). Individuals with AN also regularly report feeling isolated, alone, and cut off from others due to the experience of being misunderstood by others:

People with eating disorders are isolated and surrounded by people who don’t understand what we think or feel…Some of us need our Eds [eating disorders] still and aren’t ready to recover…We can’t go ask for safe advice from non-EDs without a risk of being hospitalized or shunned (Dias 2003, p. 37).

This feeling reflects studies indicating that people with eating disorders like AN (as well as bulimia nervosa and binge eating disorder) face *stigmatization* from the public, who tend to see individuals as responsible for their disorder (e.g., due to vanity and a desire for thinness, a lack of self-control and self-discipline, etc.) (Brelet et al. 2021). This stigmatization can also occur from those who see AN specifically as a way of garnering attention and sympathy, leading to an assumption that since it’s a “manufactured” condition it therefore ought to be easy to overcome (ibid. p.14). In addition to public stigma, people with AN also face stigma from healthcare professionals, many of whom lack the knowledge, experience, and resources to understand their experience and provide the help they need (ibid. p.15).

The stigmatization and stereotyping of anorectic individuals can result in individuals feeling as though others no longer see them as persons but merely as the *manifestation of their condition*:

I wasn’t allowed to associate with other people.... I wasn’t allowed to play sports ... so there was nothing else in my life that I was good at.... My only other identity was grades and my body.... I was always known as the skinny one (quoted in Lamoureux & Bottorff 2005, p. 175).

Everybody around me thought they knew more about it than I did. I felt like the loneliest person on the planet ... everyone was telling me what was wrong with me and how I should feel (quoted in Wooldridge 2014, p. 209).

This tendency often translates into the reduction, or even *infantilization*, of anorectic individuals into simplistic caricatures that portray them as difficult, even silly, people who do not know the harm they are causing themselves. Living with AN therefore not only transforms one’s body but also one’s interpersonal relations and sense of recognition (or lack thereof) from others (Mikhaylova and Dokuka 2022).

This persistent feeling of exclusion and nonrecognition can lead anorectic individuals to seek out alternative spaces of connection and understanding. As mentioned above, one way they do this is through the transformation of the condition into one’s trusted friend “Ana” — a friend who provides recognition by understanding one’s values, goals, desires, and who will not leave. More recently though, with the advent of digital technology, one place people increasingly turn to is online “ProAna” communities that offer a variety of resources for helping individuals develop and maintain disordered eating practices:a “ProAna” lifestyle.

Predictably, ProAna sites have been the subject of an intense media and medical backlash (e.g., Christodoulou 2012; Koszowska 2012). This resistance is fueled by the assumption that ProAna websites misrepresent AN; they portray it not as a disorder but rather as a legitimate *lifestyle choice*. And there is some truth to this. Nevertheless, the picture is more complicated than this simplistic portrayal suggests (Firkins et al. 2019). Many ProAna websites also include support and encouragement for *recovery*, as well as providing a broader sense of solidarity, belonging, and understanding for individuals who often feel lonely, misunderstood, and stigmatized (Brotsky and Giles 2007; Giles 2006; Norris et al. 2006; Smith et al. 2013).

ProAna communities become *spaces of recognition* that help combat the loneliness many feel in everyday life and give individuals an empowered sense of agency. Understanding this fuller picture of the role these spaces play in people’s lives is crucial for developing more effective intervention and treatment strategies. We lack the space for a full discussion of this idea here (see Osler and Krueger 2021), but we can note several things. As spaces of recognition, ProAna communities provide a significant sense of affective support, belonging, and comfort. Their role, in other words, is not just *epistemic* (i.e., providing useful ProAna information) but also *affective* (i.e., providing opportunities for affirmation and sharing). One study found that messages of emotional support were the most frequent form of social support found on ProAna blogs (Tong et al. 2013). Users who may initially gravitate to ProAna sites for tips and suggestions continue frequenting them for their emotional and social rewards: “I used to go to them for tips, but now I mainly go for support and giving support to others in positive ways” (quoted in Brotsky and Giles 2007, p. 100). Members of ProAna groups also express their relief at finding “true peers for the first time” (Wooldridge 2014, p. 203).

In this way, ProAna communities have the potential to empower the agent in ways that may combat painful feelings of loneliness. They provide a space in which one enjoys recognition from like-minded others, a space in which one’s agency is not fundamentally threatened by the stigma, disapproval, and antagonism that characterizes social interaction in the offline world. It is a space in which one arises as a participant in meaningful, reciprocal conversation with other persons — a domain that manifestly affords many sought-after social goods.

While ProAna communities can help combat loneliness, being part of a ProAna community often drives and sustains one’s commitment to disordered eating. Members often share expressions of support and encouragement for fasting, even enter into group fasts together. Indeed, it is common for ProAna communities to be closed groups, where entry can only be gained, and membership kept, if one is anorectic. Joining a ProAna community not only might alleviate feelings of loneliness but, in turn, help individuals sustain anorectic practices. Perhaps more than this, being a member can also work to solidify one’s *identity* as an anorectic person (Allison et al. 2021).^5^ If loneliness acts as a trigger for AN, escaping loneliness by finding solidarity and connection in ProAna spaces can work to crystallize and sustain both anorectic practices and identity.

In sum, the relationship between AN and loneliness is complex. Loneliness can prompt the start of, or the return to, disordered eating. AN can give rise to experiences of loneliness and isolation, and attempts to find community and support can encourage people to enter social spaces that both scaffold disordered eating practices and cement one’s identity as an anorectic person by providing recognition that is missing from other communities. In each of these ways, loneliness is not a constitutive feature of AN but nevertheless can drive and even exacerbate the disorder. In order to successfully — and respectfully — treat AN, therefore, it seems likely that we need to understand the key role loneliness plays in perpetuating it.

### Loneliness and Autism

According to current diagnostic guidelines, autistic spectrum disorder (ASD) is a disturbance of an individual’s ability to engage with others and the social world (American Psychiatric Association 2013; World Health Organization 2018). It spans a range of social and communicative difficulties that vary by individual. Yet, despite increased in and public awareness of autism generally, *loneliness* in autism remains under-explored. We argue that attending to experiences of loneliness helps reveal how social difficulties experienced by autistic individuals do not merely arise out of some social deficit on the part of the autistic individual but are instead *relational*. In turn, looking at autism also broadens our previous analysis of loneliness in useful ways. It shows us that nonrecognition is not simply a psychological state or even an embodied evaluative attitude. It can be realized within *material culture*, encoded within the organizational character and structures of everyday built environments in ways that withhold possibilities for reciprocity and lead to an experience of diminished agency and loneliness.

This “materialization” of nonrecognition can occur in several ways and across several dimensions. It’s worthy of more extended consideration that we can give it here.^6^ For our purposes, we simply note that it results from design decisions that are both *intentional* and *unintentional* and involve features or qualities of environments that are both *present* and *absent*.

Consider urban spaces. Urban planning has always involved designing spaces to control behavior (e.g., putting sidewalks on one side of the street but not the other to limit pedestrian access). But this is not a value-neutral process. These decisions impact different bodies in different ways (Hendren 2020). Recent discussions of so-called “hostile architecture” brings these issues into sharper relief (Rosenberger 2020). They look at design strategies that deliberately craft public spaces in ways that discourage certain uses and, in so doing, actively discourage *certain kinds of bodies* from feeling at home within them; these decisions often disproportionately impact and marginalize vulnerable populations. Representative examples include: “anti-homeless” spikes added to a surface or ledge to discourage sitting and sleeping; “anti-sleep” benches with seat dividers or armrests that prohibit bodies from stretching out; “skatestoppers”, or small metal nubs affixed to ledges and handrails, to deter skateboarding; conspicuous security cameras that encourage self-policing in public spaces; and an absence of tables, benches, or toilets in public plazas, parklands, and privately-owned spaces where people might otherwise gather.

All these design decisions are meant to regulate bodies and behavior. Some of them are intentional and involve the presence of discernible qualities or features (e.g., anti-homeless spikes or seat-dividers); others involve the intentional or unintentional absence of behavior-supporting resources (e.g., lack of benches or toilets in public plazas). Whether intentional or unintentional, and involving presence or absence, these design decisions encode forms of *nonrecognition*. For example, anti-homeless spikes and seat dividers remove possibilities for rest, comfort, and security; they make it clear that homeless bodies, who often have nowhere else to go, are not welcome to meet their basic needs within these spaces. Moreover, by removing homeless bodies from public spaces, these hostile designs render that population *less visible* to other members of the community, which further compounds problems of stigmatization and support (ibid. pp. 888–889). Again, there is much more to say about this topic. But this brief overview is sufficient for setting up some points we want to make about autistic bodies, nonrecognition, and the built environment.

Autistic people^7^ often struggle to inhabit shared forms of life with non-autistics (i.e., “neurotypicals”) (Chapman 2019). These forms of life include things like norm-governed contexts, spatial environments, customs, rituals, habits, practices, expectations, language, patterns of emotional expression and sharing, etc. — all things that neurotypicals take for granted as they move through the world and do things with others. Without access to these resources, autistic people can find it difficult to easily communicate and navigate spaces not set up to support or accommodate their ways of moving, experiencing, and expressing emotions. Social difficulties in autism, including emotional disturbances, are a focus of current research. But *loneliness* within autism remains neglected (Umagami et al. 2022). There are probably several reasons for this neglect.

First, early descriptions stressed the “powerful desire for aloneness” autistic people sometimes have (e.g., Kanner 1943, p. 249), which may have led researchers to assume that autistic people do not experience loneliness. This assumption persists today. Autistic people are regularly described as having an “empathy deficit” (Baron-Cohen and Wheelwright 2004) and “lacking awareness of others” (Chamberlain et al. 2007), and therefore assumed to have a diminished capacity for loneliness and need for connection. But when autistic people are asked to share their experiences instead of having others make assumptions about them, they do report experiencing loneliness throughout their lives (Hickey et al. 2018). For various reasons (e.g., self-regulating their emotions), solitude can be an important social coping strategy for autistic people. But requiring periods of solitude does not mean that autistic people do not experience loneliness. In fact, they do so more intensely and frequently than their non-autistic peers (Bauminger et al. 2003). Many autistic people long for social connection and are acutely aware of how pathways for connecting remain out of reach (Causton-Theoharis et al. 2009).

Another reason for this neglect may be that the experience of loneliness is often subsumed within more general discussions of social impairments in ASD, which can lead to feelings of disconnection and alienation. However, as we’ll see below, this does not do justice to the distinct phenomenology of loneliness within autism. Moreover, it overlooks the extent to which built environments can be set up in ways that create spaces of loneliness for certain groups who inhabit and move through them — including autistic people.

Autistic people regularly describe feeling a lack of access to different aspects of the social world that neurotypical people take for granted. Consider Temple Grandin’s description of her experience:

During the last couple of years I have become aware of a kind of electricity that goes on between people, which is much subtler than overt anger, happiness, or fear. I have observed that when several people are together and having a good time, then speech and laughter follow a rhythm [...] The problem is that I can’t follow this rhythm (Grandin 2006, p. 93).

Grandin here tells us that she has a sense something is going on, a kind of bodily attunement, that helps neurotypical people coordinate their behavior and intuitively understand what others think, feel, say, and do. But she cannot experience these bodily and affective rhythms herself. She is aware of their presence *via their absence*. Other autistic people say similar things (see Robledo et al. 2012).

These experiences can lead to difficulties fitting into and becoming oriented within everyday spaces. Autistic individuals regularly describe feeling cut off from ways to connect with others who might understand their experience and share their expectations. In short, they feel a persistent absence of possibilities for *reciprocity*. One person tells us that “I feel lonely a lot of the time because I always feel like I am on the outside looking in…Loneliness, for me, looks like I am in the world but can’t interact with it, almost like being a ghost” (Deaton, 2022 n.d.). Others express a similar feeling: “I got to a stage where I was so used to being mistaken by others, misinterpreted, misunderstood; I thought, well, sod it. If you’re going to misunderstand me, I’m not going to even, going to…I just closed off later on in life. I just thought, sod it” (quoted in Hickey et al. 2018 p. 363); “I cannot talk about my real experience of life to most people, because they wouldn’t understand or be interested. That makes me feel, as the saying goes, ‘lonely in a room full of people’ and I’m fed up with it” (quoted in Umagami et al. 2022, p. 10).

Some of these difficulties connecting may involve *perceptual* differences: difficulties perceiving the expressive style, or qualitative character, of gestures, facial and emotional expressions, actions, etc., which convey important social information and help guide our social interactions (Bader and Fuchs 2022; Krueger 2021b; Rochat et al. 2013). Some of these difficulties may also involve a mismatch of *communicative* expectations: the norm-governed timing, character, and context-appropriate content of communicative exchanges. For example, an autistic person may take longer to respond to questions or verbal prompts than neurotypicals expect; conversations may be punctuated by delays and long silences — something acceptable within autistic communities — leading neurotypicals to change the subject or leave the conversation (Leary and Donnellan 2012). Or autistic people may provide a direct and unfiltered response to questions like “Do I look good in this shirt?” (“No, you do not!”) when the questioner is actually seeking affirmation. Finally, they may not make eye contact when speaking — again, common and accepted within autistic communities — and therefore be interpreted by neurotypicals as suspicious or dishonest (Chapman 2019, p. 430). These mismatches can lead autistic people to feel as though their distinctive ways of experiencing the world and talking about it are not recognized by the people they routinely encounter, creating barriers that lead to a sense of diminished agency and loneliness (Bauminger et al. 2003). The possibilities for reciprocity and recognition others take for granted are experientially present via their absence.^8^

However, this feeling of nonrecognition and diminished agency may also flow from broader *structural* issues. To return to an earlier quote, it’s not just the *people* but also the *room* that can lead to experiences of loneliness. This is because nonrecognition can be encoded within features, spatial arrangements, and design decisions of the *built environment*; the non-inclusive structure and character of these spaces are what make them spaces of loneliness for autistic people. As Sarah Ahmed observes, spatial environments — which encompass both people as well as norm-governed things and practices — function as “affective containers” (Ahmed 2014, p. 224). And as affective containers, built environments can simultaneously accommodate certain kinds of bodies and open up possibilities for emotional connection and reciprocity while, at the same time, excluding these possibilities from others.

To see how so, we can again turn to first-person narratives. Autistic people routinely describe feeling that to be an autistic person in a predominantly neurotypical world is to be a “stopped body.”^9^ What this means is that very often, autistic bodies are prohibited from extending into and taking shape within the spaces they inhabit — spaces designed to accommodate the needs, values, and patterns of movement, experience, and expression characteristic of neurotypical bodies. This is not just the case in face-to-face interaction, as we saw above. This “stopping” also occurs when autistic bodies run up against constraints built into the spatial-material environment, too. Autistic bodies try to extend into these spaces and feel them resist and push back — a form of *material* non-recognition.

For example, a noisy, brightly lit lecture hall, restaurant, pub, or retail space may negatively impact an autistic person’s auditory and visual hypersensitivity in ways neurotypical bodies don’t understand or fully appreciate. For autistic individuals, the design of these spaces does not afford feeling at home. Instead, they are disorienting and bodily upsetting; the bright lights, temperature, smells, cramped arrangement, and constant unpredictable noise make it impossible to settle comfortably into them, and instead place autistic bodies in a reactive mode where they are constantly battling against this onslaught of sensory information.

From a neurotypical perspective, it may be easy to dismiss how perceptually disorienting such environments can be for bodies with different sensory processing needs. But consider some first-person reports which remind us that “autism affects everything all the time”, as one individual puts it (Leary and Donnellan 2012, p. 56). Something as common as a glass dropped onto a wooden table can be experientially overwhelming:

I am aware that on paper it sounds about as awful as when you bite into a banana and it isn’t quite ripe enough yet. Not ideal but certainly not painful? However, for me it hurts as much as someone hitting me across the face with no warning…Noise is really painful, especially when I don’t realise it is about to happen…I would rather the person threw the glass directly at my face than drop it by mistake (Henderson 2020).

Within a public setting, the acoustics, unpredictability, and sheer number of different sound sources at a given time can intensify this effect. Consider this description of early experiences of school environments:

I was often lazy in school because sometimes me ears distorted the teacher’s instructions or my eyes blurred to stop me seeing the blackboard properly. Or sometimes I would hear a word or two at the start and understand it and the next lot of words sort of merged into one another. And I could not make head or tail of it. I can now recall that one could sometimes refer to my vision and hearing as being like an untuned television (quoted in Leary and Donnellan 2012, p. 56).

Another person tells us that, when navigating public spaces, “I still become visually overloaded…If I could get away with going around blindfolded there are times when that would be easier than being distracted by a bunch of visual clutter (quoted in ibid. p.57). Still another reports how sounds, smells, tactile qualities, and visual aspects coalesce into an oppressive and inescapable atmosphere: “It is like a constant blanket of sound that just keeps coming at you until you are totally disoriented” (quoted in Boldsen 2022a, p. 5).

As Boldsen (2022a) observes, autistic bodies can easily become destabilized within these spaces that are not set up to accommodate their distinctive sensory needs, leading to a sense of powerlessness and diminished agency. What is normally a tacit part of the environment for neurotypicals — background noise in the auditory and visual environment — for autistic people “loses its character as unnoticed background and instead moves to the foreground of experience” (ibid. p. 6). This destabilization makes it difficult to navigate the built environment; it constricts the individual’s sense of agency, leading them to withdraw as a means of self-regulation. But it also has social significance, too. This is because “the sense of presence in the concrete interaction dissolves into a sea of noise and movement”, an intensifying saturation of experience that “leads to an experiential disconnection from the perceptual situation and its meaning” (ibid. p.6). As a result, sensory environments can disrupt the autistic individual’s sense of familiarity with others and the security they might otherwise feel in situations and spaces that, instead of supporting them, instead seem to actively work against them. Indeed, such experiences can cement the feeling that the world others easily inhabit is not a shared world for them, that they are out of place, underscoring a pervasive sense of somehow being “different”, even being “wrong”.

There is more to say, of course.^10^ But these narratives (and many more like them) indicate that experiences of absence, diminished agency, and nonrecognition at the heart of loneliness can arise because, for many, specific parts of the world are not set up to facilitate easy movement, connection, and sharing. In the case of autism, built environments — and the norm-governed practices that surround them — are generally set up to support neurotypical styles of being in the world. While these environments may not have been intentionally designed to exclude autistic bodies, their *absence* of accommodation for different sensorimotor needs is telling. These environments therefore often fail to facilitate autistic ways of sensing, moving, and relating to others; they reflect a structural mismatch between the needs, interests, experienced salience, and felt possibilities between the autistic person and the wider social and material environment (Strijbos 2022). In this way, nonrecognition can be materialized within the structures of everyday environments. And by impeding autistic styles of embodiment, these environments can, for some, become spaces of loneliness.

A final point. What this analysis also highlights is that the lack of social understanding autistic people often experience is not *unidirectional*, that is, exclusively on the side of the autistic person. Neurotypical individuals also struggle to understand and recognize autistic individuals (McGeer 2009). This relational perspective unsettles the idea that autistic individuals experience loneliness mainly due to social impairments on their side, which undermine their ability to successfully access social goods. Instead, it highlights ways that autistic individuals experience loneliness because their distinctive ways of socially interacting and being in the world are not sensitively responded to, or accommodated by, neurotypicals (Chapman 2019). To put it another way, social goods seem not just out of reach but rather *placed out of reach* by social norms and environments designed exclusively for neurotypicals. Loneliness is not a necessary feature of what it is to be autistic; nor does it drive autism. Rather, loneliness arises out of contingent factors that govern norms of social interaction and social spaces that exclude and ostracize autistic persons.

## Conclusion

Loneliness comes in many degrees and textures. We can experience a fleeting moment of loneliness or chronic longer-term episodes; we can experience loneliness due to contingent features of our current situation beyond our control, or as somehow connected with a more essential part of our self-identity. As our three case studies have shown, the theme of loneliness threads its way through psychopathology. We might therefore think that loneliness is, as van den Berg states, at the heart of all psychopathologies. However, we have sought to show that while loneliness might be common to many, if not all, psychopathologies, we must nevertheless attend to *specific ways* in which loneliness manifests in *specific conditions*. Doing so not only captures experiential differences between the loneliness of, say, depression, anorexia nervosa, and autism — thus enriching our understanding of each — but importantly, this perspective also reveals *how* and *why* loneliness arises in distinctive ways.

Recognizing loneliness within psychopathology isn’t just descriptively useful in that it provides a richer phenomenological profile of depression, anorexia nervosa, and autism. It may have practical significance, too, insofar as it impacts diagnostic and remedial strategies. First, simply talking more openly about loneliness can provide individuals — whatever their current level of mental health and wellbeing — with both a shared vocabulary for articulating their experience to others, as well as a descriptive vocabulary that helps them recognize what they’re feeling *is*, in fact, loneliness. Stigmatizing or denying loneliness may push individuals to further isolation, which can exacerbate their loneliness. Conversely, developing public narratives about loneliness may decrease its negative impact by driving public acceptance and motivating individuals to both acknowledge and act on their feelings.

In the context of psychopathology, it may also help craft more nuanced intervention strategies. To take just one example, consider AN. Foregrounding how stigmatization and loneliness in everyday life drives many anorectics to seek out recognition in alternative communities, such as online ProAna communities, emphasizes the extent to which therapeutic interventions should be tailored to address individuals’ *relational* needs, that is, their desire for community, connection, and acceptance (Firkins et al. 2019). But this is often not done within current treatment protocols. Since AN is often conceptualized as primarily a *cognitive* disorder (i.e., stemming from an individual’s faulty beliefs about their body, a desire for thinness, etc.), Cognitive Behavioral Therapy is often recommended to change these faulty beliefs and recalibrate behavior. And for some, these cognitive strategies may be effective. But they are also limited. They overlook the full picture given by first-person reports of how many anorectics live with and actively maintain AN as a *shared project* (Kuhle 2019, p. 120), which includes descriptions of the important role online communities often play in sustaining this shared work. As we discussed above, narratives of people living with AN often emphasize the emotional and affective components of this project — “…I mean, I wouldn’t say anorexia is a thought as such…it’s more of a feeling I, I WANT to do it and I guess in a way it’s almost an EMOTION, anorexia” (Charland et al. 2013) — and many emphasize the emotional and social support they get form ProAna sites (Tong et al. 2013). Intervention strategies should therefore take these reports as their starting point.

Consequently, this relational approach — which, again, emphasizes the centrality of loneliness and desire for connection in driving AN — reminds us that when encouraging individuals to move away from ProAna sites as part of their recovery process, we are asking them to do more than just modify some core beliefs. We are asking them to leave a *community* — an online space that meets different aspects of their basic needs for connection, affiliation, and sharing. And this is crucial because many with AN feel regularly misunderstood by families, friends, and doctors (Stinson 2019). One individual diagnosed with AN tells us that “a lot of psychiatrists don’t know what they’re talking about…it shouldn’t be looked at on the surface [i.e., just in terms of beliefs]” (quoted in Malson 1998, p. 2155). So, clinicians should familiarize themselves with these communities in order to recognize how they meet these relational needs — a familiarity that will, ideally, lead to more refined therapeutic practices.

For example, a gradual withdrawal from ProAna communities, instead of summarily cutting off all contact with them as a first step towards recovery, may be a more effective strategy (Osler and Krueger 2021). For many, leaving these communities is a transitional process, one that is not complete until other alternatives are found. Acknowledging the role of loneliness in driving AN can help us see how so. And exploring experiences of loneliness in relation to other psychopathologies will, in this way, likely reveal yet more ways in which loneliness can be experienced in relation to various disorders and conditions, and suggest additional pathways for treatment, intervention, and social connection.

Finally, this focus on loneliness in psychopathology also highlights the importance of addressing issues of recognition and reciprocity when it comes to loneliness work more generally. When we are lonely, we feel that we are not seen — and often, powerless to do anything about it. Accordingly, whether in the context of psychopathology or elsewhere, we should be mindful that loneliness and nonrecognition is a problem involving broader social *and* material factors, and work to alleviate loneliness should be tailored accordingly.

## Footnotes

1 See McHugh Power et al. (2018); Motta (2021); Seemann (2022).

2 John McGraw (1995) similarly characterizes loneliness as an experience of absence (i.e., of the desired other’s presence), perceived, he tells us, “as a presence, albeit a presence that is felt in the manner of an absence, that is, as a privation or deprivation of the other” (p.44).

3 Drawing upon Hannah Arendt, Lucas (2019) argues persuasively that loneliness is a structural element of totalitarian regimes insofar as they attempt to eradicate individual freedom, creativity, and political agency in the service of consolidating and preserving the power of the state. See also Wilkinson (2022) for a feminist perspective on the politics of “structural” loneliness, as she terms it, in the context of late capitalism.

4 Not always, of course. Some forms of reciprocity can be inauthentic, as in the example above (i.e., individuals conveying their indifference and nonrecognition by a superficial or obviously performative adherence to basic social norms). But one way reciprocity can be said to signal recognition is when the former is expressive of a certain kind of *emotional stance*, “one which is held by the recognizing subject and directed toward the other person” (Jardine 2020 p. 315). Such a stance, according to Jardine, directly expresses an affective evaluation of the (recognized) other by signaling an openness to engage with them in ways acknowledging their individuality, significance, and moral status. In short, it signals that they are *seen*. See Jardine (2020) for further details of this emotional stance.

5 For a detailed discussion of AN and identity, see Schiff (2022).

6 We are grateful for an anonymous reviewer pressing us for more clarity on this point.

7 We here follow terminological preferences of neurodiversity proponents who endorse identity-first language (“autistic persons”) to emphasize the connection between cognitive and emotional styles and selfhood (Pellicano and Stears 2011).

8 Additional support for this view comes from studies showing that autistic people report interactions with other autistic persons to be efficient and pleasurable even when they struggle to connect and communicate with neurotypicals (Schilbach 2016). One individual tells us: “If I socialize with other Aspergians of pretty much my own functionality, then all of the so-called impairments simply don’t exist…we share the same operating systems, so there are no impairments” (Cornish 2008 p. 158).

9 For more detailed discussions of this idea, see Krueger (2021a, c). The notion of “stopped bodies” is drawn from Ahmed’s (2007) critical phenomenological analysis of stopped bodies in the context of race and queerness. See also Brancazio (2020) for a rich attempt to think through some of these issues from the perspective of ecological psychology and enactivist frameworks.

10 For a more extensive discussion of these and related themes, see Boldsen (2022a, b).
